# The effect of cone beam computerized tomography voxel size and the presence of root filling on the assessment of middle mesial canals in mandibular molar teeth

**DOI:** 10.1007/s00784-024-05773-5

**Published:** 2024-06-25

**Authors:** Tansu Çimen, Salih Düzgün, İpek Eraslan Akyüz, Hüseyin Sinan Topçuoğlu

**Affiliations:** 1 Department of Oral and Maxillofacial Radiology, Faculty of Dentistry, Alanya Alaaddin Keykubat University, Antalya, Turkey; 2 Department of Endodontics, Faculty of Dentistry, Erciyes University, Melikgazi, Kayseri, 38039 Turkey

**Keywords:** Cone-beam computed tomography, Middle mesial canal, Voxel size

## Abstract

**Introduction:**

The study aims to compare the detection of the middle mesial canal (MMC) in mandibular molar teeth using cone beam computed tomography (CBCT) with different voxel sizes when the mesiobuccal (MB) and mesiolingual (ML) canals have three distinct phases (unpreparation, preparation and obturation and the removal of the obturation and repreparation).

**Methods:**

Two hundred forty-two extracted human mandibular molars were collected and kept in a physiological saline solution prior to use. 0.2-, 0.28- and 0.35-mm voxel sizes CBCT (*n* = 242) were performed in three phases (Ph): Ph1, no MB and ML canal preparation or obturation; Ph2, after MB and ML canals preparation and obturation; and Ph3, after the removal of the obturation of MB and ML canals and canals repreparation. Images were analyzed using OnDemand3D® software. After the CBCT acquisition in Ph3, all the samples were clarified to visualize the presence of the MMC directly. A blinded, previously calibrated examiner analyzed all the images.

**Results:**

The MMC was detected in 15 of the 242 teeth after the clearing technique. The lowest MMC detection rate was observed at 0.35-mm voxel size regardless of the ML and MB canal condition, while the highest was observed at 0.2-mm voxel size (*P* < 0.05). There is no statistically significant difference between 0.2-mm and 0.28-mm voxel sizes (*P* > 0.05). In all voxel sizes, the highest rate of detectability of the MMC was seen in Phase 1, while the lowest was in Phase 2.

**Conclusions:**

It may be appropriate to take a 0.20-mm voxel size CBCT image, especially after the removal of root canal filling.

**Clinical relevance:**

An appropriate CBCT voxel size and the absence of root canal filling in the root canal system help to detect the missing MMC.

## Introduction

Endodontic treatment aims to prevent or heal apical periodontitis. The ultimate clinical objective of root canal treatment is the three-dimensional obturation of the endodontic spaces subsequent to them being wholly cleaned, shaped, and disinfected [1]. However, the complexity of the root canal anatomy can negatively affect the outcome of root canal treatment. Detecting the presence of additional root canals can prevent unfavorable results in terms of root canal treatment. This include, insufficient root canal shaping and cleaning, and the inability to eliminate bacteria from infected root canals [2]. Therefore, it is important to know the internal anatomy of teeth for successful root canal treatment [3].

Mandibular molar teeth usually have two roots, the mesial and the distal. The mesial root commonly presents one mesiobuccal (MB) and one mesiolingual (ML) canal; nevertheless, there are reports in the literature of there being variant anatomical configurations of mandibular molars There are some reports of mandibular first molars with 5, 6, or even 7 canals [4, 5]. In addition, many reports show the presence of a third canal - the middle mesial canal (MMC) - in the mesial root of mandibular first molars [6–8]. In the literature, the rate of MMC detection varies between 0.26% and 46.15% [8, 9].

Many studies describe the root canal morphology of mandibular molar teeth [10–13]. Methods such as root canal staining and tooth clearing [13], conventional and digital radiography [14], radiographic assessment enhanced with contrast medium [15] and micro-computed tomography (micro-CT) [16] have been used to analyze canal morphology. In recent years, cone beam computed tomography (CBCT) has been an essential device for diagnosing and planning endodontic treatments due to its ability to provide accurate three-dimensional views of the internal anatomy of teeth [17, 18].

Although CBCT has some advantages, it also has some disadvantages. The presence of scattering artefacts can adversely affect scan quality [19]. Materials such as root canal filling material, intracanal medicaments such as calcium hydroxide [20], and intracanal metallic posts frequently used in endodontic treatment, can also compromise artefact formation [19, 21, 22].

In a previous study, Vizotto et al. [23] evaluated the effect of different voxel sizes and the presence of canal filling on the frequency of MB2 canal detection in maxillary molars on CBCT examination. However, when the literature is reviewed, there is no study evaluating the effects of varying voxel sizes and the presence of root canal filling in the determination of the MMC of mandibular molar teeth. Therefore, the current study aims to compare the ability of CBCT with different voxel sizes to detect the MMC in mesial roots of mandibular molars when the MB and ML canals have three distinct phases (Ph1, no MB and ML canal preparation or obturation; Ph2, after MB and ML canal preparation and obturation; and Ph3, after the removal of obturation of MB and ML canals and canal repreparation).

## Materials and methods

### Study design

Two hundred and forty-two extracted human mandibular molars were collected from people between the ages of 18–60 and kept in a physiological saline solution prior to use. 2D radiography was taken from the teeth before study. Teeth which had a completely formed apex and no previous root filling, resorption, or calcifications were included, while teeth which had open apex, previous root filling, resorption, or calcifications were excluded. The presence of an MMC of mandibular molar teeth was evaluated in three distinct phases as follows:

Phase 1 (Ph1): – no MB and ML canal preparation or obturation.

Phase 2 (Ph2) – after MB and ML canal preparation and obturation.

Phase 3 (Ph3) – after the removal of MB and ML canal obturation and canal repreparation.

### Root canal preparation

Endodontic access cavities were opened using diamond-coated burs (Diatech; Coltene Whaledent, Altststten, Switzerland) under water cooling. Each mesial canal’s working length (WL) was determined by visualizing a #10 K-file (Maillefer Instruments, Ballaigues, Switzerland) through the apical foramen and subtracting 1 mm. Teeth with apical diameters larger than the #10 K-file were excluded. Mesiobuccal and mesiolingual canals were shaped using OneCurve (Micro-Mega SA Besancon Cedex, France) file [(25/0.06)] at WL as recommended by the manufacturer. After each preparation, the mesial canals were irrigated using 3 mL sodium hypochlorite (NaOCl). After completion of the preparation, the canals were irrigated with 5 mL 17% EDTA for one minute and subsequently rinsed with 5 mL distilled water.

### Root canal obturation

All canals were obturated with only a master gutta-percha (size 25, 0.06 taper) and AH Plus sealer (Dentsply Sirona) using a single cone technique. AH Plus was introduced into the root canal utilizing a Lentulo spiral. Mesiodistal and buccolingual radiographs were taken to confirm complete filling. After root obturation, the coronal 1 mm filling material was removed, and the endodontic access cavity was filled with a temporary filling material (Cavit; 3 M ESPE, Seefeld, Germany). All specimens were then stored at 37^0^ C and 100% humidity for two weeks to allow for the complete setting of the sealer.

### Removal of root canal obturation

The ProTaper universal retreatment (Dentsply Sirona) system with D1, D2, and D3 files was used to remove root canal filling material. The D1 file for the coronal third, the D2 file for the middle third, and the D3 file for the apical third were used with light pressure until the WL was reached. After the root canal filling material was completely removed from the mesial canals, the root canals were reprepared again using a OneCurve file [(25/0.06)] at WL. After each preparation, the mesial canals were irrigated using 3 mL sodium hypochlorite (NaOCl). After completion of the preparation, they were irrigated with 5 mL 17% EDTA for one minute, and subsequently rinsed with 5 mL distilled water. All files were used according to the manufacturer’s instructions.

Canal preparation, root canal obturation, removal of root canal obturation and canal re-preparation were performed by a single operator.

### Evaluation of the presence of the MMC

We scanned 242 teeth at the voxel value (0.2 mm) with the highest probability of detecting MMC. After analyzing the CBCT images so obtained, we were able to detect the presence of MMC in only 13 of the 242 teeth. After CBCT acquisition, the same 242 teeth were clarified to visualize the presence of the MMC directly. The external surface of each sample except coronal 3 mm was covered with three layers of nail varnish. Indian ink (Royal Talens, Apeldoorn, Netherlands) was placed into the pulp chamber. The samples were placed in a vacuum chamber for six hours and then rinsed in tap water for six hours to remove the excess dye. The nail varnish was removed with a surgical blade. The samples were then kept in 5% hydrochloric acid for 12 h, washed in tap water (4 h) and dehydrated in an ascending concentrating of alcohol (75, 85, 95, 100) (3 h each), and immersed in methyl salicylate for clarification (24). After the clearing technique, we were able to definitively detect the presence of MMC in 15 of the 242 teeth.

**Fig. 1:**
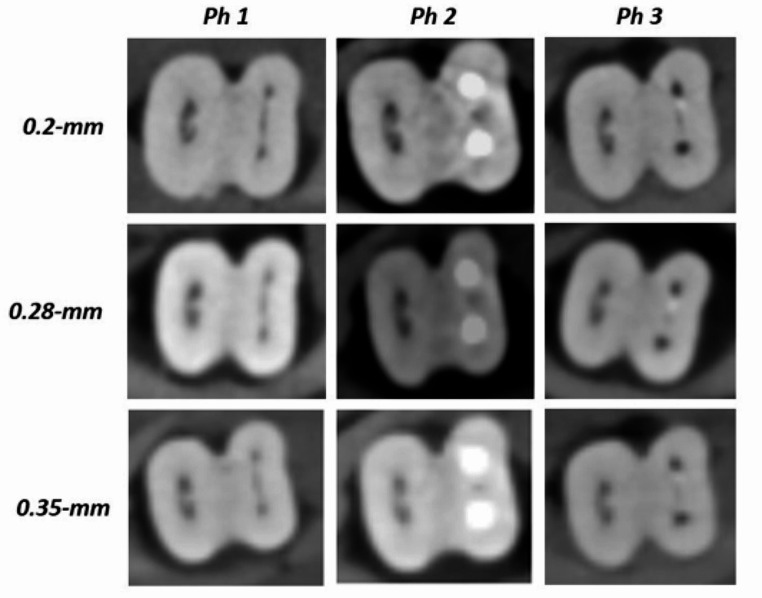
Different CBCT voxel sizes of a sample in Phase 1 (Ph1): – no MB and ML canals preparation or obturation; Phase 2 (Ph2) – after MB and ML canals preparation and obturation; Phase 3 (Ph3) – after MB and ML canals obturation removal and canals repreparation

**Fig. 2:**
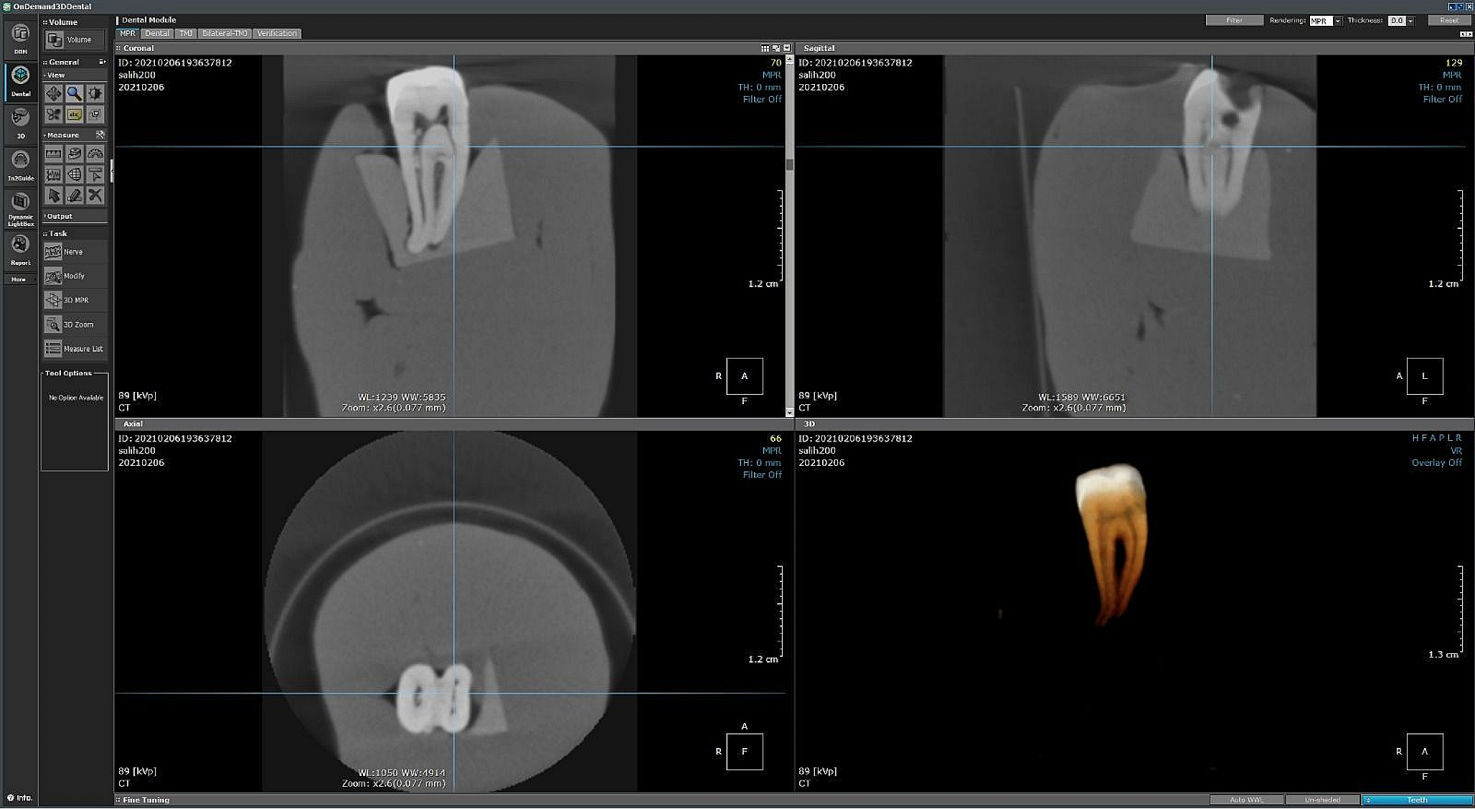
The representative image which analyzed using the OnDemand3D® software

After the clearing technique, 15 teeth with the definite presence of MMC were analyzed for Ph1, Ph2 and Ph3 at different voxel sizes using CBCT. The roots of each vertically positioned tooth were placed into a block of bone to simulate the mandible. A layer of wax was applied over the external bone surface to simulate soft tissues. For the tomographic acquisition at Ph1, Ph2, and Ph3, groups of 5 teeth were placed together on the desk of the KaVo OP 3D Pro tomography device (Kavo Dental GmbH, Biberach/Riss, Germany). Axial, frontal, and sagittal sections were obtained by following specific protocols based on the voxel resolution: 0.2-mm voxel (5 × 5 cm field of view [FOV], 1.2-s, 3.2 mA, 90 kVp, 34 mGycm^2^), 0.28-mm voxel (8 × 8 cm FOV, 1.2-s, 3.2 mA, 90 kV, 76 mGycm^2^) and 0.35-mm voxel (8 × 15 cm FOV, 2.3-s, 3.2 mA, 90 kV, 146 mGycm^2^) (Fig. 1). 15 teeth were scanned with CBCT by a radiologist. Images were analyzed using the OnDemand3D® software by two endodontists (CyberMed, Seoul, Republic of Korea)(Fig. 2).

### CBCT imaging data and statistical analysis

Observer calibration was achieved by evaluating the presence of MMC in 20 CBCT images that were not included in the current study. The same observation using the 20 CBCT images was repeated after a 15-day interval. When the observers detected the presence of MMC in the teeth they were assessing, they recorded this. In cases where the two observers disagreed, the observers made a joint assessment and reached a consensus. Kappa statistics were used to check for intra- and inter-observer reproducibility. The Kappa values of intra- and inter-observer agreement were 0.80 and 0.70, respectively. Chi-square test was used to determine differences in the identification of MMCs between voxel sizes. SPSS v.20 for Windows (SPSS, Chicago, IL, USA) was used for all statistical analyses, with the level of significance set at 5% (*p* < 0.05).

## Results

MMC was detected in 15 of the 242 teeth after the clearing technique. The number of MMC detections for each root situation following clearing technique regarding voxel sizes were as shown in Table 1. The lowest MMC detection rate was observed at 0.35 mm voxel size, regardless of the ML and MB canal condition, while the highest MMC detection rate was observed at 0.2-mm voxel size (*P* < 0.05). There is no statistically significant difference between the 0.2-mm and 0.28-mm voxel sizes (*P* > 0.05). The ML and MB canal condition affected MMC detectability at all voxel size and phases, with the exception Ph 2 and 3 with regard to the 0.35-mm voxel size. In all voxel sizes, the highest MMC detectability rate was seen in Ph 1, while the lowest was seen in Ph 2. In the case of Ph 1, there was no statistically significant difference between 0.2-mm and 0.28-mm voxel sizes in terms of MMC detectability (*P* > 0.05), while the detection rate of MMC in 0.35-mm voxel size was statistically significantly lower than the 0.2-mm and 0.28-mm voxel sizes (*P* < 0.05). In Ph 2, there was no statistically significant difference between all voxel sizes in terms of MMC detectability (*P* > 0.05). In Ph 3, there was no statistically significant difference between both the 0.2-mm voxel size and 0.28-mm voxel size, and the 0.28-mm voxel size and 0.35-mm voxel size in terms of the detectability of MMC (*P* > 0.05), while there was a statistically significant difference between 0.2-mm voxel size and 0.35-mm voxel size (*P* < 0.05). The MMC detection rate was statistically significantly higher in Ph 1 compared to Ph 2 and Ph 3 when all voxel sizes were evaluated (*P* < 0.05), while there was no significantly different between Ph2 and Ph3 (*P* > 0.05).

**Table 1:** The numbers and percentage of MMC detection of 15 teeth detected after clearing technique according to each root situation and each voxel sizes

	Ph 1	Ph 2	Ph 3
**0.2-mm voxel**	13/15 (87%) ^a, A^	4/15 (27%) ^b, C^	6/15 (40%) ^b, D^
**0.28-mm voxel**	13/15 (87%) ^c, A^	3/15 (20%) ^d, C^	4/15 (27%) ^d, D, E^
**0.35-mm voxel**	7/15 (47%) ^e, B^	1/15 (7%) ^f, C^	1/15 (7%) ^f, E^

*Phase 1 (Ph1): no MB and ML canals preparation or obturation; Phase 2 (Ph2) – after MB and ML canals preparation and obturation; Phase 3 (Ph3) – after MB and ML canals obturation removal and canals repreparation

*MMC- Middle mesial canal

* Different lowercase letters in one same line and different uppercase letters in one same column indicate statistically significant difference

## Discussion

Anatomical complexities, such as additional root canals, can play an important role in the outcome of root canal treatments [24]. Therefore, undetected and untreated MMC jeopardizes treatment success [2]. The current study assessed the ability of CBCT with different voxel sizes to detect the presence of MMC canals under various MB and ML canal conditions.

The clearing technique was preferred in the current study, both because this was successfully used in the methodology of a previous study [23] and because micro-CT is expensive. Since the current study is the first study on this subject, power analysis could not be performed for the sample size. This can be considered as a limitation of the study.

CBCT is often preferred because of its positive contribution to the diagnosis and/or management of complex endodontic problems [25, 26]. CBCT is also an important diagnostic tool for detecting MMC clinically [6, 27]. CBCT provides more accurate imaging information for diagnosing endodontic diseases and conditions and detecting MMC. Still, no evidence exists with regard to the influence of root filling and voxel size in MMC canal detection.

CBCT is an important diagnostic tool for investigating the presence of MMC after especially unsuccessful root canal treatment. However, the radiation dose that the patient will be exposed to should be considered when CBCT imaging is required. It is known that the amount of radiation which the patient will be exposed to is directly related to the voxel size. Therefore, the dental professional should consider image acquisitions that provide both adequate diagnostic ability and also involve reduced radiation exposure in line with ALARA principle [28].

Although CBCT is an important diagnostic tool, the sclerosed and/or accessory anatomy may not be easily identified where CBCT has a poor resolution [29]. Many of these technical parameters such as FOV, voltage peak (kVp) and filtration, anode current (mA), exposure time, and rotation arc influence the image quality as well as the radiation dose to the patient [30]. Voxel size affects the resolution of CBCT images. A small voxel size is needed to obtain a high-resolution CBCT image [31]. Almost all CBCT devices scan with a smaller FOV using a smaller voxel size, resulting in higher spatial resolution images. A smaller FOV also reduces the amount of scattered radiation. This reduces image noise and contributes to improved image quality [30]. In the current study, three different voxel sizes were used depending on the difference in exposure parameters. In the current study the 0.2-mm voxel size had the highest resolution, while the 0.35-mm voxel size had the lowest resolution. The radiation dose was lowest at 0.2-mm voxel size and highest at 0.35-mm voxel size. In order to obtain the voxel sizes used in the study, the CBCT device (KaVo OP 3D Pro tomography device) we have available used automatically provided us with the technical parameters since it used an automatic exposure control mechanism. Therefore, the fact that FOV values and exposure time are not the same, is a limitation of this study.

In the current study, MMC was clearly detected in 0.2-mm and 0.28-mm voxel size images at Ph 1, while the detection of MMC in 0.35-mm voxel size images was reduced by half. The detectability rate of MMC in 0.2-mm and 0.28-mm size images decreased dramatically at Ph2; additionally, only a MMC in the case of one tooth was detected at 0.35-mm voxel size images at Ph 2. The detection rate of MMC in 0.2-mm and 0.28-mm voxel sizes increased again in Ph 3 compared to Ph 2, but the detection rate of MMC in 0.35-mm voxel size remained the same when passing from Ph 2 to Ph 3. The reason for the difference in MMC detectability when all phases are evaluated among themselves can be that the presence of gutta-percha within the canal can cause radiographic artifacts that can interfere with the visibility of additional root canals during the examination of CBCT images. The reason why the detection rate of the MMC at 0.35-mm voxel size does not change when passing from Ph 2 to Ph 3 may be due to the lower resolution of the 0.35-mm voxel size compared to other voxel sizes. Another reason may be that after the preparation and filling of root canals in Ph2, the effect of a radiographic artifact caused by gutta percha increases due to the decrease in the distance between ML and MB canals and MMCs. In addition, in all phases, the lowest detectability rate of MMC was found in 0.35-mm voxel size images. This may be due to the decrease in image resolution as the voxel size increases.

According to the results of the current study, the maximum detection rate of MMC was obtained in 0.2-mm voxel size images in all phases. However, there was no statistical difference between the 0.2-mm voxel size images and the 0.28-mm voxel size images. Thus, the patient was exposed to less radiation at 0.2-mm voxel size compared to 0.28-mm voxel size, while the presence of MMC was also detected at a similar rate. In addition, when MMC is suspected in lower molar teeth with unsuccessful root canal treatment, it would be more appropriate to take a CBCT image after the removal of the root canal obturation.

More studies with larger sample sizes using CBCT devices which have adjustable technical parameters are needed.

## Conclusion

Within the limits of this study, when MMC is suspected in mandibular teeth with unsuccessful root canal treatment, it may be appropriate to take a 0.2-mm voxel size CBCT image especially after root canal filling removal because of its greater accuracy and lower radiation exposure in line with the ALARA principle.
